# Targeting *Cis*-Regulatory Elements for Rice Grain Quality Improvement

**DOI:** 10.3389/fpls.2021.705834

**Published:** 2021-08-11

**Authors:** Yu Ding, Jiannan Zhu, Dongsheng Zhao, Qiaoquan Liu, Qingqing Yang, Tao Zhang

**Affiliations:** ^1^Key Laboratory of Plant Functional Genomics of the Ministry of Education, Jiangsu Key Laboratory of Crop Genomics and Molecular Breeding, Jiangsu Co-Innovation Center for Modern Production Technology of Grain Crops, Jiangsu Key Laboratory of Crop Genetics and Physiology, College of Agriculture, Yangzhou University, Yangzhou, China; ^2^Department of Biotechnology, School of Life Science and Technology, University of Electronic Science and Technology of China, Chengdu, China

**Keywords:** rice, *cis*-regulatory element, genome editing, grain quality, upstream open reading frame

## Abstract

Rice is the most important source of food worldwide, providing energy, and nutrition for more than half of the population worldwide. Rice grain quality is a complex trait that is affected by several factors, such as the genotype and environment, and is a major target for rice breeders. *Cis*-regulatory elements (CREs) are the regions of non-coding DNA, which play a critical role in gene expression regulation. Compared with gene knockout, CRE modifications can fine-tune the expression levels of target genes. Genome editing has provided opportunities to modify the genomes of organisms in a precise and predictable way. Recently, the promoter modifications of coding genes using genome editing technologies in plant improvement have become popular. In this study, we reviewed the results of recent studies on the identification, characterization, and application of CREs involved in rice grain quality. We proposed CREs as preferred potential targets to create allelic diversity and to improve quality traits *via* genome editing strategies in rice. We also discussed potential challenges and experimental considerations for the improvement in grain quality in crop plants.

## Introduction

Rice provides major nutrients and energy for more than half of the population worldwide. In the last 30 years, rice yield has been continuously improved through implementing a series of breeding programs (Xu et al., 2020). Meanwhile, the demand for high quality, multiresistance, and wide adaptability of rice variants is also increasing, especially for the high-quality rice under conditions of improved living standards (Rao et al., 2014). Generally, rice grain quality is a combination of milling, appearance, eating, cooking, nutritional, and hygiene traits (Zhou et al., 2020). Therefore, to meet the needs of consumers and producers, researchers have to understand the molecular mechanisms and genetic basis that determines rice quality, and breeders and seed companies have to develop rice varieties with excellent quality and high yield. However, the direct regulation of coding genes for target traits is difficult and is often accompanied by negative effects; therefore, it cannot achieve the expected effect. For instance, Pérez et al. (2019) used CRISPR/Cas9 to introduce mutations affecting the *Wx* gene [encoding granule-bound starch synthase I (GBSSI)] in the rice endosperm. The amylose content (AC) declined to as low as 5% in homozygous seeds, accompanied by abnormal cellular organization in the aleurone layer and amorphous starch grain structures. Plant genetic engineering efforts to improve grain quality in crop plants using well-characterized promoter elements to modify the expression of regulatory genes and/or transcription factors (TFs) have proven to be advantageous (Yang et al., 2019). Therefore, in this perspective, we mainly discussed the identification and analysis of *cis*-regulatory elements (CREs) involved in rice grain quality and the modification of CREs *via* genome editing technologies for the improvement in rice grain quality.

## Genetics and Genomics of Rice Grain Quality

In general, four main quality traits, namely, milling properties, appearance, nutritional value, and cooking quality, are widely used to assess rice grain quality (Zhou et al., 2020). With the rapid development of high-throughput technologies and functional genomics, many genes controlling important quality traits have been cloned in rice, and some molecular mechanisms have been characterized (Zhou et al., 2020). Milling quality is a complex grain trait, including the recovery of brown, milled, and head rice. Several major quantitative trait loci (QTLs) are associated with rice milling quality, such as *qBRR-10*, which influence brown rice recovery (Ren et al., 2016). Rice grain shape is an important index of appearance quality and yield, and several related genes and TFs have been studied (Huang et al., 2013). For example, *GS3, GW5, GLW7, GW8, GS2*, and *GS9* are the main genes or transcription regulators affecting variations in rice grain shape and size. In addition, genes such as *GL7/GW7, GL3.1, GS5, GW2*, and *GL3.3*, which were identified in natural variants, have profound effects on grain shape and functions, and their mechanisms have been studied (Huang et al., 2013). The composition and structure of starch play crucial roles in rice grain quality, especially for eating and cooking quality. Enzymes involved in the starch synthesis pathway (including GBSS, SS, SBE, ISA, and PUL) and associated TFs (i.e., *Dull, OsEBP89, OsRSR1*, and *OsbZIP58*) have been clearly determined in rice (Zhou et al., 2020). Many major functional genes have been identified, which affect nutrients such as seed proteins, essential amino acids, vitamins, minerals, and anthocyanins (Das et al., 2020). Similarly, grain quality-related genes and their metabolic pathways have been studied in other crops, and the results have been discussed in a recent review (Birla et al., 2017). The characterization of these quality-related genes has laid the foundation for the improvement in rice quality. Although many genes related to crop grain quality have been explored, and their genetic mechanisms have been analyzed, how these genes can be better used in breeding production and commercial utilization requires further study.

## *Cis*-Regulatory Elements and Their Mining in Plants

Fundamentally, in addition to genes, many elements present in the plant genome control the gene expression levels *via* interactions with DNA or regulatory proteins. Limited genetic diversity restricts the amount and effectiveness of the improvement in rice quality. Recent results showed that genetic changes in CREs of genes play important roles in shaping phenotypic diversity by altering gene expression (Swinnen et al., 2016).

### *Cis*-Regulatory Elements: Important Regulators of Gene Expression

The CREs are the noncoding DNA containing binding sites for TFs or other regulatory molecules that affect transcription, and they ultimately guide plant growth and development, cell differentiation, and responses to various stresses (Lu et al., 2018). Therefore, the whole genome identification and functional characterization of CREs involved in the DNA–protein interactions is a key aspect to understand plant transcription regulation (Lu et al., 2018). Generally, CREs in eukaryotes include promoters, enhancers, and other CREs, among which enhancers tend to be much more variable (Meng et al., 2021). Enhancers that drive transcription are independent of their distance and location from their cognate promoters, which allows a gene to be regulated by multiple remote enhancers with different spatiotemporal activities (Meng et al., 2021). The sequence structure of promoters of quality-related genes is relatively simple and conserved, and their functions have been clarified in rice. The identification and functional analysis of CREs are also in progress in rice (Swinnen et al., 2016). More comprehensive collections and analyses of CREs in rice are necessary, which will accelerate the fine-tuning of the improvement in rice quality *via* CRE editing.

### Mining CREs Based on “Omics”

The systematic identification of CREs in plant genomes is critically important to understand the transcriptional regulation and its exploitation for the improvement in quality. Some important results have been obtained using approaches involving sequence conservation within short distances from target genes (Huang et al., 2020a). Additionally, some databases, such as PlantCARE, which store all plant transcription sites, consensus sequences, and matrices described in the literature, can also be used to predict possible CREs for one or more genes (http://bioinformatics.psb.ugent.be/webtools/plantcare/html/). Several limitations are as follows: sequence conservation can be restricted, CREs that are far from their target genes will not be detected, and we have less information regarding their tissue specificity and functionality (Lu et al., 2018).

Generally, CREs are preferentially located in the accessible chromatin regions (España et al., 2017). Therefore, genomics and epigenomics are used to identify and analyze CREs. For example, the alignment of the upstream sequences of *OsPLD*α*1* orthologs across 34 rice accessions revealed sequence variations and identified CREs involved in differential transcription of orthologs, which resulted in the low expression of *OsPLD*α*1* and reduced free fatty acid content in the oil, facilitating good quality bran oil (Kaur et al., 2020). A genome-wide association study (GWAS) screen of 45 natural accessions and the *pif4* mutant in *Arabidopsis* identified CREs affecting *SAUR26* gene expression (Wang et al., 2021). Similar CRE mining strategies have been reported in rice (Ho and Geisler, 2019). The whole genome transcriptome profiling using microarrays was employed to discover CREs associated with drought and salinity stress tolerance in rice (Mishra et al., 2018).

In addition, CREs can be identified based on their elevated sensitivity to enzymes such as the bacterial transposase Tn5, DNase I, and micrococcal nuclease (MNase) (Zhang et al., 2016; Lu et al., 2017; Zhao et al., 2020). The coupling of chromatin accessibility assays with the high-throughput DNA sequencing, such as DNase-seq, MNase-seq, self-transcribing active regulatory region sequencing (STARR-seq), and assay for transposase-accessible chromatin using sequencing (ATAC-seq), represents an important technological development and has enabled the identification of CREs on a genome-wide scale (Lu et al., 2018). Numerous putative CREs were identified in plant species through the strategies of genetic, epigenomic, and functional molecular characterization (Lu et al., 2019). A major limitation to these assays is the lack of cell-type resolution and the paucity of information regarding which CREs function in specific tissues or cell types at the genome-wide scale, especially in endosperm tissue, which is closely related to rice quality.

Moreover, ribosome profiling (ribo-seq) provides a viable strategy to analyze active translation by determining ribosome occupancy in a transcriptome-wide manner, which can then be used to identify valid upstream open reading frames (uORFs), a type of CRE (Lulla et al., 2019). However, the large-scale identification of uORFs has not been reported in rice. Thus, ribo-seq is expected to be an effective tool to identify rice quality-related uORFs. Furthermore, combined with the multiomics data, such as RNA sequencing (RNA-seq), chromatin immunoprecipitation sequencing (ChIP-seq), and proteomics, CREs and their related TFs could be mined more accurately (Farmer et al., 2021).

## The Key Roles of *Cis*-Regulatory Elements in the Improvement in Rice Quality

Unlike changes to protein-coding genes, which often result in easily interpretable loss-of-function alleles, the mutations of CREs offer the potential of fine-tuning gene expression without other adverse effects, leading to improved rice quality (Huang et al., 2020a; Zeng et al., 2020). Therefore, more attention should be paid to CREs and their influence on gene expression.

Employing both bioinformatic and experimental methods, CREs involved in gene regulation have been identified, and most of the genes affected by these CREs have been found to encode TFs that regulate plant development (España et al., 2017). Several universal CREs have been identified in the 5′ upstream region of the starch synthetase gene, lysine metabolism genes, and seed-storage protein genes in rice and/or other crops (Table 1) (Chen et al., 2012). Recent studies have shown that CREs play an important role in improving rice quality traits (Table 1).

**Table 1:** Improvement in rice quality achieved *via* the modification of *cis*-regulatory elements.

**Target trait**	**Gene**	**Target function**	***Cis-*elements-dependent regulation**	**CRE**	**CRE core sequence**	**CRE location**	**Modification**	**Target gene expression pattern**		**References**
								**Gene expression**	**Tissue or organ**	
Cooking and eating quality	*Wx*	Granule-bound starch synthase I	Apparent amylose content	unknown element, A-box, CAAT-box, Endosperm-box	TATAATAAT, GGCCAATCT	Upstream, Intron	Genome editing	Downregulation	Developing endosperm	Huang et al., 2020a; Zeng et al., 2020
Nutritional quality	*OsbZIP18*	Basic leucine zipper transcription factor	Branched-chain amino acids levels	/	/	Upstream	Natural variation	Upregulation	Leaves	Sun et al., 2020
	*OsBCAT1*	branched-chain aminotransferase1	Branched-chain amino acids levels	ACE element	/	Upstream	Genome editing	Upregulation	Leaves	Sun et al., 2020
	*OsBCAT2*	branched-chain aminotransferase2	Branched-chain amino acids levels	C-box *cis*-element	GTCA	Upstream	Genome editing	Upregulation	Leaves	Sun et al., 2020
	*OsAAP6*	Amino acid transporter	Grain protein content	copper-responsive element, Inr-element, sulfur-responsive element	/	Upstream	Natural variation	Diversity^#^	Endosperms	Peng et al., 2014
	*OsGluA2*	Glutelin type-A2 precursor	Grain protein content	BIHD1OS	/	Upstream	Natural variation	Diversity	Endosperms	Yang et al., 2019
	*REP-1*	Cysteine proteinase	Glutelin degradation	GA-responsive element	TAACAGA, TAACGTA, CAACTC	Upstream	Deletion and point-mutation	Upregulation	Seeds	Sutoh and Yamauchi, 2003
	*Kala4*	bHLH transcription factor	Anthocyanin production	/	/	Upstream	Natural variation	Upregulation	Leaves	Oikawa et al., 2015
	*OsPLDα1*	Lipolytic enzyme	Free fatty acid content and flavor	ARFAT element, SEBF element	TGTCTC, TTGTCTC	Upstream	Natural variation	Downregulation	Immature grains	Kaur et al., 2020
	*Chalk5*	Vacuolar H^+^-translocating pyrophosphatase	Grain chalkiness	RY/G-box, CACT tetranucleotide	CATGCA, CACT	Upstream	Natural variation	Downregulation	Endosperms	Li et al., 2014
Appearance quality and yield	*GS5*	Serine carboxypeptidase	Grain size	ABA-responsive element	/	Upstream	Natural variation	Upregulation	Developing seeds	Li et al., 2011; Xu et al., 2015
	*qSH1/RPL*	BEL1-type HomeoBox gene	Seed shattering	RY-repeat	/	Upstream	Natural variation	Upregulation	Abscission layer	Konishi et al., 2006
	*Ghd7*	CCT domain protein	Grain number	/	/	Upstream	Natural variation	Diversity	Young stem and leaves	Lu et al., 2012, Xue et al., 2008
	*GW8/SPL16*	SBP-domain transcription factor	Grain shape, quality, and size	/	/	Upstream	Natural variation	Downregulation	Developing panicles	Wang et al., 2012
	*GW7*	TONNEAU1-recruiting motif protein	Grain shape and quality	GTAC motif	GTAC	Upstream	Natural variation	Upregulation	Young panicles	Wang et al., 2015
	*FZP*	APETALA2/ETHYLENE response factor	Grain width and weight	CACTA transposon	CACTA	Upstream	Natural variation	Downregulation	Young panicles	Wang et al., 2020
	*GW6*	Gibberellin-regulated GAST family protein	Grain size	CAAT-box	CACACAAATCT	Upstream	Natural variation	Upregulation	Young panicles	Shi et al., 2020
	*OsACBP2*	Acyl-CoA-binding protein	Seed development and size	Skn-I-like motif	GTCAT	Upstream	Deletion mutation	Downregulation	Seeds	Guo et al., 2019
	*Eui1*	GA-deactivating enzyme	Plant height, grain yield	RY motif-containing *cis*-silencing element	CATGCA	Intron	T-DNA insertion mutagenesis	Upregulation	Young panicles	Xie et al., 2018
	*OsREM20*	B3 domain transcription factor	Grain number per panicle	CArG box-containing inverted repeat	CATTAATTAG	Upstream	Natural variation/Genome editing	Upregulation	1-week-old seedlings	Wu et al., 2021
	*GSE5*	Plasma membrane-associated protein	Grain size	/	/	Upstream	Natural variation	Downregulation	Spikelet hulls	Duan et al., 2017
	*GLW7/SPL13*	plant-specific transcription factor OsSPL13	Grain shape and yield	/	/	5′-UTR	T-DNA insertion mutagenesis	Upregulation	Panicles and florets	Si et al., 2016
	*TGW2*	Cell number regulator	Grain width and weight	/	/	Upstream	Natural variation	Upregulation	Glumes	Ruan et al., 2020
	*OsMADS1*	CW domain-containing zinc finger protein	Grain width	CATTTC motif	CATTTC	Upstream	Genome editing	Downregulation	Young panicles	Huang et al., 2020b

# *Represents diversity in the regulation region of the target gene*.

The ARFAT and SEBF elements have been identified as CREs that might act as repressors in regulating *OsPLD*α*1* expression, which lead to decreased free fatty acid content in oil and improve the flavor and quality of rice bran oil (Table 1) (Kaur et al., 2020). Two common variations in the potential CREs of the *OsAAP6* 5′-untranslated region (5′-UTR) seem to be associated with grain protein content diversity and nutritional quality, mainly in *indica* cultivars (Peng et al., 2014). A single nucleotide polymorphism (SNP) located in the *OsGluA2* promoter region is associated with its transcript expression level and grain protein content diversity (Yang et al., 2019). For amino acids, natural variations in the *OsbZIP18* promoter contribute to branched-chain amino acid levels in rice (Sun et al., 2020). Two consensus nucleotide polymorphisms in the *Chalk5* promoter in rice varieties might partly account for the differences in *Chalk5* mRNA levels that contribute to natural variation in grain chalkiness (Li et al., 2014). *GRAIN WIDTH 7* (*GW7*) is an important gene that controls cell division in the spikelet hulls, and its expression is regulated by the repressive TF, i.e., *GRAIN WIDTH8* (*GW8*). A mutation in the CRE in the promoter of *GW7* led to enhanced *GW7* expression and ultimately to the improved yield and grain quality (Sakamoto and Matsuoka, 2008; Wang et al., 2015). Similarly, natural variation in the promoter of *GSE5* contributes to grain size diversity, and that in *TGW2* determines rice grain width and weight (Duan et al., 2017; Ruan et al., 2020). A novel variation in the *FRIZZLE PANICLE* (*FZP*) gene promoter improved rice grain number and yield (Wang et al., 2020).

## Upstream Open Reading Frames: Important *Cis*-Regulatory Elements in the 5′ Leading Sequence

Upstream open reading frames, as translational regulatory elements, are located in the 5′-UTR of eukaryotic mRNAs, and generally inhibit the translation initiation of downstream primary ORFs (pORFs) through ribosome stalling (Kurihara, 2020). In plants, uORFs have been predicted in ~30% of the 5′-UTRs of genes, and some of these uORFs have been reported to regulate crucial growth and developmental processes (von Arnim et al., 2014). Increasing numbers of excellent crop genes have been identified and characterized (Zhou et al., 2020). Furthermore, to improve crop characteristics, many genes that regulate important traits are required to have a high translation rate, rather than their functional loss or reduction (Xu et al., 2017; Reis et al., 2020). Based on the knowledge that uORFs negatively affect the translation of the pORF, the strategy of modulating uORFs to fine-tune the translation could be used to analyze gene function and improve crop traits. For example, uORF TBF1-mediated translation enabled engineered *Arabidopsis* and rice broad-spectrum disease resistance without any reduction in grain yield (Xu et al., 2017).

## Genome Editing Toward Better Grain QualitY *via* Targeting *Cis*-Regulatory Elements

All the earlier studies suggested that CRE modification can regulate the expression of key genes for rice quality and effectively improve grain quality. However, the identification of natural mutations in the promoter regulatory regions of the gene is time-consuming and difficult, which slows the improvement in rice quality. Recently, genome editing technology has been developed and optimized and has been applied successfully to a large number of plants, which has accelerated the identification and application of regulatory elements in gene promoter regions in rice (Huang et al., 2020a; Zeng et al., 2020). Genome editing is a versatile, relevant, and preferred technique for functional genomics, as well as crop improvement, involving introducing DNA mutations in the form of deletions and/or insertions or base substitutions in target gene sequences (Fiaz et al., 2019; Tabassum et al., 2021).

The starch synthase gene *Wx* is very important for rice eating and cooking quality; therefore, it has been a popular target for study. Zeng et al. (2020) disrupted the Endosperm-box, A-box, and CAAT-box of the promoter sequence and intron region of the *Wx* gene using genome editing, which generated new *Wx* alleles producing various ACs by quantitative regulation of its expression. Novel *Wx* alleles, in which CREs in the *Wx* promoter near a predicted TATA-box were edited using the CRISPR/Cas9 system, produced fine-tuned amylose levels and improved the rice grain quality (Huang et al., 2020a). The deletion mutants of the CATTTC motif exhibited the lower expression of *OsMADS1* and produced narrower rice grains (Huang et al., 2020b). Wu et al. (2021) enhanced rice grain production by manipulating the CArG box-containing inverted repeat sequence of *OsREM20*. Although there are only a limited number of studies demonstrating successful editing of CREs for crop improvement, it is anticipated that genome editing techniques such as CRISPR/Cas9 will lead to further CRE editing to improve rice grain quality.

For uORFs, editing the uORF of *LsGGP2* increased oxidative stress tolerance and the ascorbate content by ~150% in lettuce (Zhang et al., 2018). In *Arabidopsis*, a conserved peptide uORF (CPuORF33) was identified in the 5′-UTR of *AtHB1* mRNA, which ensures a relatively low level of *AtHB1* expression in aerial parts and avoids adverse phenotypes (Ribone et al., 2017). Similarly, there are several studies on the regulation of uORFs in morphogenesis, signaling pathways, and nutrient absorption stress response in *Arabidopsis* (Zhang et al., 2020). Reis et al. (2020) identified the *PHO1* uORF in the genomes of crops such as rice, maize, barley, and wheat, which improved plant growth under inorganic phosphorus (Pi)-deficient conditions. A tandem repeat sequence in the 5′-UTR of *GLW7* alters its expression by affecting transcription and translation, resulting in enhanced rice grain length and yield (Si et al., 2016). Hence, uORFs have great potential to improve rice grain quality by positively regulating target genes.

## Conclusion and Future Perspectives

**Figure 1:**
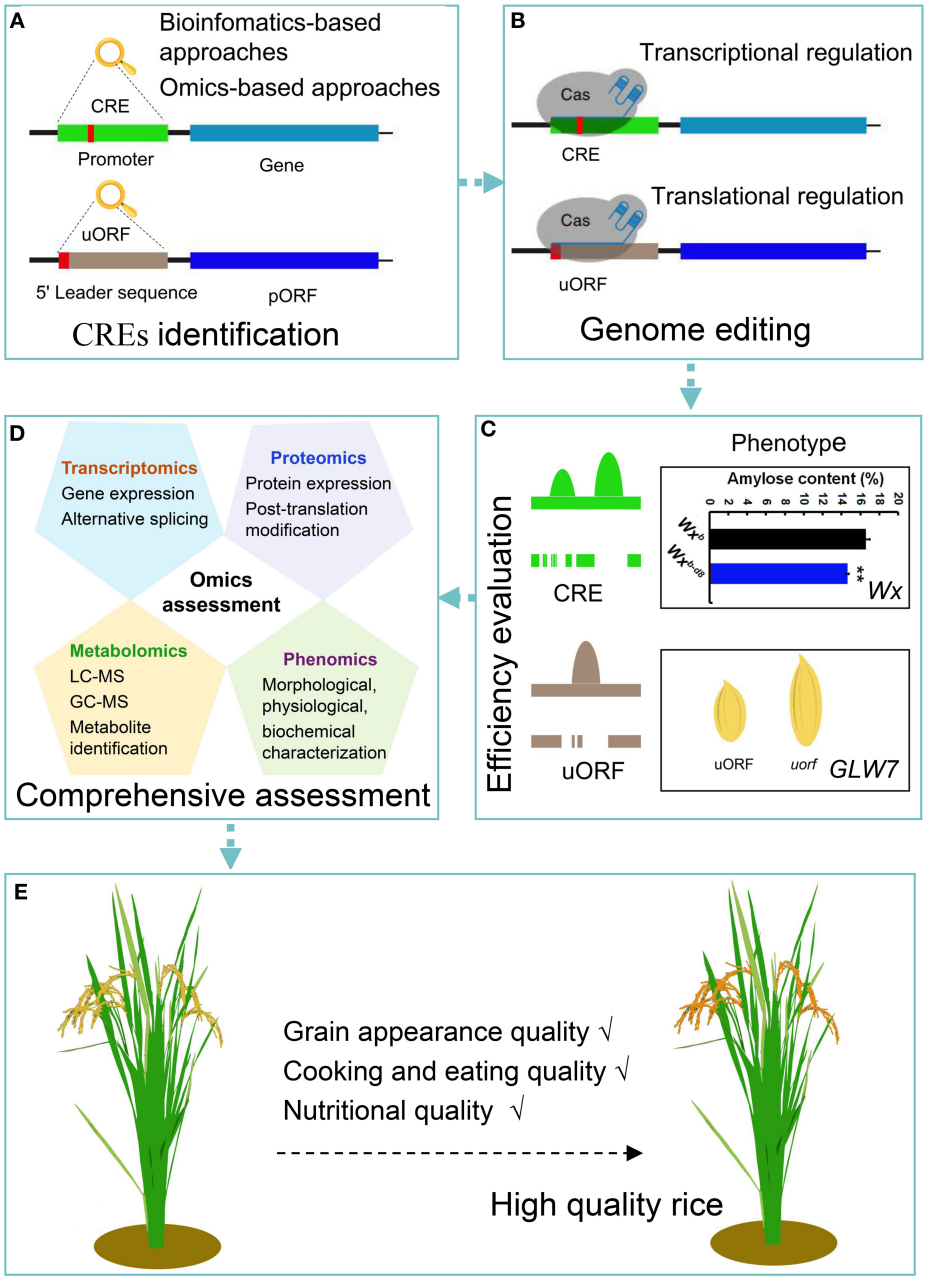
Schematic representation of the workflow depicting the application of genome editing approaches to obtain high grain quality rice. **(A)** CREs are identified by multiomics strategies and bioinformatic approaches. *Cis*-regulatory sequences are linear nucleotide fragments of non-coding DNA. Their localization and orientation in relation to genes and activity vary (Verter and Botha, 2010). uORFs, as translational regulatory elements, are located in the 5′ leader sequence of eukaryotic mRNAs and generally inhibit the translation initiation of downstream pORFs (Kurihara, 2020). **(B)** Selection of the desired target DNA sequences of CREs and uORFs, and recognition of PAM sequences for genome editing, which regulates gene expression at the transcription and translation levels, respectively. **(C)** Efficiency evaluation using molecular identification and phenotypic analysis. For example, a genome editing strategy was used to edit the core promoter region of the *Wx* gene, and the obtained *Wx*^*b*−*d*8^ mutant had a nine nucleotide deletion (Huang et al., 2020a). In the *Wx*^*b*−*d*8^ mutant, the predicted core promoter region was disrupted, which decreased *Wx* expression compared with the wild-type *Wx*^*b*^ and reduced the amylose content (%) in the mature rice seeds (Huang et al., 2020a). The same method is applicable to edit the predicted uORF of *GLW7* (Si et al., 2016). The deletion of predicted uORF in the *GLW7* 5′ leader sequence causing increased expression levels of the GLW7 protein and enhanced rice grain length. Colored peaks represent different TF-binding events within CREs or uORFs, and peak height indicates the chromatin accessibility from genomic data sets (i.e., DHase-seq and ATAC-seq). Lines with spaces beneath the TF-binding peak indicate destroyed CRE or uORF sequences. **(D)** Comprehensive assessment of edited rice using multiomics strategies. **(E)** Obtaining the desired high-quality rice. CRE, *Cis*-regulatory element; uORF, upstream open reading frame; pORF, primary open reading frame; PAM, protospacer adjacent motif; *Wx*, the *Waxy* gene encoding granule-bound starch synthase I, which controls amylose synthesis in rice endosperm; *Wx*^*b*^, Nipponbare carrying the *Wx*^*b*^ allele; *Wx*^*b*−*d*8^, the *Wxb-d8* mutant with nine nucleotide deletion of the core promoter region in Nipponbare (*Wx*^*b*^). GLW7, which is encoding the plant-specific TF OsSPL13, positively regulates cell size in the grain hull, resulting in enhanced rice grain length and yield (Si et al., 2016); TF, transcription factor.

The aim of the plant functional genomics is to explore the key genes in plant growth and development, to determine their regulatory mechanisms, and to fine-tune the gene expression effectively, with the aim of improving traits for research and/or commercial use. Crop quality is an important trait whose further improvement requires increased research resources. Recently, several crop quality-related genes and metabolic pathways have been identified and explored (Zhou et al., 2020). In addition, the high-throughput technologies have accelerated the identification and analysis of these functional quality-related genes (Hernandez-Garcia and Finer, 2014). However, it is difficult to achieve the desired improvement in quality through the direct manipulation of these elite genes. The identification and modification of the CREs of these genes would provide an appropriate approach to modulate their expression (Huang et al., 2020c). The popularity of genome editing further confirms that CREs are good potential targets to create new alleles for the improvement in rice quality in transgene-free derivatives (Figure 1).

Recently developed omics approaches, such as DNase-seq, ATAC-seq, and ribo-seq, have identified certain CREs and uORFs. Thus, a more comprehensive analysis of CREs in promoter regions and uORFs in 5′ leading sequences will increase opportunities for quality-related genome editing. Additionally, these techniques are complex and have limitations, especially in the study of CREs and uORFs related to endosperm traits in rice. Hence, the development of an effective and simple method or system for the CRE identification in rice seeds is essential to improve rice quality. Moreover, CREs are very short (usually only a few nucleotides), and constructing a uORF mutant requires the start codon to be modified; therefore, more precise genome editing techniques should be considered to avoid limited protospacer adjacent motif (PAM) sites, off-target mutations, and low homology-directed repair (HDR) efficiency.

In addition to the technological issues, there are significant gaps in our knowledge of gene regulation in most species. At present, studies on CREs and uORFs have mainly focused on their identification and functional analysis (Hernandez-Garcia and Finer, 2014). Given the importance of CREs for gene expression in crops, in-depth studies, such as CRE regulatory mechanisms and related metabolic connections, require further research. Thus, the analyses of the CREs related to crop quality and their transcriptional and translational regulatory modifications are essential. It is hoped that the combination of CREs and genome editing technologies will enable the simultaneous manipulation of multiple traits in rice (Figure 1).

Moreover, it is not easy to forensically detect genome editing events at the molecular level, especially as no foreign DNA exists in the line in which the regulatory element(s) are subtly edited. Given this limitation, the downstream “omics” technologies that can reveal the effects of the edits, such as proteomics and metabolomics, should be considered to fully assess the changes of proteins and/or their compositions in novel foodstuffs from the edited crops. The integration of the in-depth understanding of gene regulatory mechanisms and related networks, and genome editing to identify and modify CREs at the single nucleotide level in plant genomes, might represent a promising strategy for future crop improvement.

## Author Contributions

QY, YD, and TZ organized and wrote the manuscript. JZ collected and summarized some points. QL and DZ provided critical evaluation and edited the text. All the authors have read and approved the manuscript.

## Conflict of Interest

The authors declare that the research was conducted in the absence of any commercial or financial relationships that could be construed as a potential conflict of interest.

## Publisher's Note

All claims expressed in this article are solely those of the authors and do not necessarily represent those of their affiliated organizations, or those of the publisher, the editors and the reviewers. Any product that may be evaluated in this article, or claim that may be made by its manufacturer, is not guaranteed or endorsed by the publisher.

## Abbreviations

ARFAT: auxin response factor
*AtHB1*: *Arabidopsis thaliana* HomeoBox 1
FZP: Frizzle panicle
GBSS: Granule-bound starch synthase
*GL3*.1: Grain length 3.1
*GL3.3*: Grain length 3.1
*GLW7*: Grain length and weight on chromosome 7
*GS2*: Grain size on chromosome 2
*GS3*: Grain size on chromosome 3
*GS5*: Grain size on chromosome 5
*GS9*: Grain shape on chromosome 9
*GSE5*: Grain size on chromosome 5
*GW2*: Grain width 2
*GW5*: QTL for grain width and weight on chromosome 5
*GW7*: Grain width 7
*GW8*: Grain width 8
ISA: Isoamylase
*LsGGP2*: GDP-l-galactose phosphorylase 2
*OsAAP6*: Amino acid permease 6
*OsbZIP18*: basic leucine zipper transcription factor 18
*OsbZIP58*: basic leucine zipper transcription factor 58
*OsEBP89, Oryza sativa* ethylene-responsive element binding protein: clone 89
*OsGluA2*: glutelin type-A2 precursor
*OsMADS1*: MADS-domain transcription factor
*OsPLD*α*1*: Phospholipase D alpha 1
*OsREM20*: *Oryza sativa* REPRODUCTIVE MERISTEM 20
*OsRSR1*: Rice starch regulator 1
*OsSPL16*: *squamosa* promoter binding protein-like 16
*PHO1*: PHOSPHATE1
PUL: pullulanase
*qBRR-10*: Brown rice rate on chromosome 10
*qSH1*: QTL of seed shattering in chromosome 1
*SAUR26*: SMALL AUXIN UP RNA gene 26
SBE: starch-branching enzyme
SEBF: silencing element binding factor
SS: soluble starch synthase
*TGW2*: 1000-grain weight 2.
